# Supplementary material for: Intra-abdominal hematomas and identifiable risk factors in patients receiving subcutaneous enoxaparin
Source: PLoS One. 2024 Apr 16;19(4):e0301906. doi: 10.1371/journal.pone.0301906 (PMC11020830; doi:10.1371/journal.pone.0301906)
Supplement: Attachment — Submitted filename: PLUS ONE Responses to Reviewers.docx [file pone.0301906.s002.docx]

**Responses to Reviewers**

Academic Editor

1. “Each CT scan was reviewed personally by a single physician to confirm clinically significant bleeding to ensure relevant cases were identified.”

Is the physician one of the authors? Yes, the physician that again read the CT scan to confirm the previous reading is also an author. This section was modified to make it transparent to the reader (see below).

Each CT scan was initially read by a staff radiologist with documentation of the bleed in the electronic medical record. At study enrollment, each CT was again reviewed personally by a single physician also, the author, to confirm clinically significant bleeding to ensure relevant cases were identified.


2. “With COVID-19 patients receiving intermediate or therapeutic dose anticoagulation, Interleukin – 6 blockade, steroids, all increases the risk for major bleeding”

Please elaborate on this

This section was expanded and revised a little especially in regards to the number of positive covid-19 patients we had; however, there is still a lot of unknowns in this area that do not allow us to elaborate more at this time.

3. What was the lovenox dose used for morbidly obese with a BMI of 30 kg/m2?

The section regarding enoxaparin dosing was modified and addressed the dosing in morbidly obese patients.

Enoxaparin prophylactic dosing consisted of 40mg SQ daily unless the patient creatinine clearance (CrCl) was less than or equal to 30ml/min at which the dosing was reduced to 30mg SQ daily. Enoxaparin treatment dosing consisted of 1mg/kg SQ twice daily unless the patient creatinine clearance was less than or equal to 30ml/min at which the dosing was reduced to 1mg/kg SQ daily. Actual body weight was used for patient dosing and Body Mass Index (BMI) was not taken into consideration with alternative dosing.

Reviewer 1

1. Is the manuscript technically sound, and do the data support the conclusions?

The manuscript must describe a technically sound piece of scientific research with data that supports the conclusions. Experiments must have been conducted rigorously, with appropriate controls, replication, and sample sizes. The conclusions must be drawn appropriately based on the data presented.

In our opinion, the data does support our conclusions and is sound based on our analysis; however, the research would be more robust if it were a prospective with a statistical analysis. With the limited number of cases and the financial constraints to conduct a prospective study, we conducted a retrospective review to begin to answer the question is the side effect of intra-abdominal hematomas more common than that which is published in the literature to date.

Reviewer #1: Yes

Reviewer #2: Yes

Reviewer 2

2. Has the statistical analysis been performed appropriately and rigorously?

No because as above, with the limited number of cases and the financial constraints to conduct a prospective study, we conducted a retrospective review to begin to answer the question is the side effect of intra-abdominal hematomas more common than that which is published in the literature to date and we believe our results do answer that question and can raise awareness among providers when caring for patients treated with enoxaparin.

Reviewer #1: No

Reviewer #2: Yes

Reviewer 3

3. Have the authors made all data underlying the findings in their manuscript fully available?

The patient data has been deidentified and will be sent with the original manuscript and will support the findings summarized in our research article.

The [PLOS Data policy](https://protect-us.mimecast.com/s/qIz6CkRKnzUAN5kyuNq5Za?domain=plosone.org) requires authors to make all data underlying the findings described in their manuscript fully available without restriction, with rare exception (please refer to the Data Availability Statement in the manuscript PDF file). The data should be provided as part of the manuscript or its supporting information, or deposited to a public repository. For example, in addition to summary statistics, the data points behind means, medians and variance measures should be available. If there are restrictions on publicly sharing data—e.g. participant privacy or use of data from a third party—those must be specified.

Reviewer #1: Yes

Reviewer #2: Yes

Reviewer 4

4. Is the manuscript presented in an intelligible fashion and written in standard English?

PLOS ONE does not copyedit accepted manuscripts, so the language in submitted articles must be clear, correct, and unambiguous. Any typographical or grammatical errors should be corrected at revision, so please note any specific errors here.

Our article was reviewed by the authors again with grammatical and formatting changes and then sent to another reviewer and researcher for feedback on proofing and editing.

Reviewer #1: Yes

Reviewer #2: Yes

Reviewer 5

5. Review Comments to the Author

Please use the space provided to explain your answers to the questions above. You may also include additional comments for the author, including concerns about dual publication, research ethics, or publication ethics. (Please upload your review as an attachment if it exceeds 20,000 characters)

Reviewer #1: This study provides crucial insights into enoxaparin-associated abdominal hematomas, emphasizing risk factors such as age, renal impairment, and obesity. Despite its retrospective design and limited sample size, the findings align with existing literature, contributing valuable knowledge to guide clinicians in cautious enoxaparin dosing.

Reviewer #2: The authors did great job looking into the incidence of abdominal hematoma after enoxaparin use. This is a retrospective study, which have a few limitations and well mentioned in the discussion. It would be great to add more information about the concurrent presence or absence of COVID-19 infections in those patients included in analysis. It would appropriate to add year after November at one place. Overall, it is well written.

We did go back and review our patients again to determine how many were affected by the Covid-19 pandemic. Upon review, 3 of our patients were positive with 8 patients testing negative and the remaining 8 were not tested on his or her hospital admission. These details were added to the manuscript.

Reviewer 6

6. PLOS authors have the option to publish the peer review history of their article ([what does this mean?](https://protect-us.mimecast.com/s/E815ClYLoAim7X1wTj45Md?domain=journals.plos.org)). If published, this will include your full peer review and any attached files.


If you choose “no”, your identity will remain anonymous but your review may still be made public.


**Do you want your identity to be public for this peer review?** For information about this choice, including consent withdrawal, please see our [Privacy Policy](https://protect-us.mimecast.com/s/Qa_fCmZ6pBt82WpocLX3dM?domain=plos.org).

Reviewer #1: **Yes:** Ripudaman Munjal

Reviewer #2: No
